# Epidemiology of *Citrobacter* spp. infections among hospitalized patients: a systematic review and meta-analysis

**DOI:** 10.1186/s12879-024-09575-8

**Published:** 2024-07-02

**Authors:** Pérince Fonton, Nasreen Hassoun-Kheir, Stephan Harbarth

**Affiliations:** Infection Control Program, Geneva University Hospitals and Faculty of Medicine, WHO Collaborating Center, Rue Gabrielle-Perret-Gentil 4, CH-1205 Geneva, Switzerland

**Keywords:** *Citrobacter* spp. infections, Hospitalized patients, Nosocomial infections, Carbapenem resistant – 3rd generation cephalosporin resistant, Outbreak

## Abstract

**Background:**

Infections due to *Citrobacter* species are increasingly observed in hospitalized patients and are often multidrug-resistant. Yet, the magnitude and burden of *Citrobacter* spp. resistance in the hospital setting have not been reported. We aimed to evaluate the epidemiology of *Citrobacter* spp. infections among hospitalized patients, their main resistance patterns and *Citrobacter* spp. involvement in hospital outbreaks.

**Methods:**

We conducted a systematic review and meta-analysis of published literature (PROSPERO registration Jan-2023, CRD42023390084). We searched Embase, Medline and grey literature for studies on hospitalized patients diagnosed with *Citrobacter* spp. infections, and nosocomial outbreaks due to *Citrobacter* spp. published during the years 2000–2022. We included observational, interventional, surveillance studies and outbreak reports. Outcomes of interest were the frequency of *Citrobacter* spp. infections among hospitalized patients and 3rd generation cephalosporin and/or carbapenem resistance percentages in these infections. We used random-effects models to generate pooled outcome estimates and evaluated risk of bias and quality of reporting of outbreaks.

**Results:**

We screened 1609 deduplicated publications, assessed 148 full-texts, and included 41 studies (15 observational, 13 surveillance and 13 outbreak studies). *Citrobacter* spp. urinary tract- and bloodstream infections were most frequently reported, with *Citrobacter freundii* being the main causative species. Hospital-acquired infection occurred in 85% (838/990) of hospitalized patients with *Citrobacter* infection. After 2010, an increasing number of patients with *Citrobacter* spp. infections was reported in observational studies. Pooled frequency estimates for *Citrobacter* spp. infections could not be generated due to lack of data. The pooled prevalence of ESBL and carbapenemase producers among *Citrobacter* isolates were 22% (95%CI 4–50%, 7 studies) and 18% (95%CI 0–63%, 4 studies), respectively. An increased frequency of reported *Citrobacter* outbreaks was observed after 2016, with an infection/colonization ratio of 1:3 and a case-fatality ratio of 7% (6/89 patients). Common outbreak sources were sinks, toilets, contaminated food and injection material. Implemented preventive measures included environmental cleaning, isolation of positive patients and reinforcement of hand hygiene. Only seven out of 13 outbreaks (54%) were definitively controlled.

**Conclusion:**

This review highlights the clinical importance of endemic and epidemic *Citrobacter* spp. in healthcare settings. As an emerging, multidrug‑resistant nosocomial pathogen it requires heightened awareness and further dedicated surveillance efforts.

**Supplementary Information:**

The online version contains supplementary material available at 10.1186/s12879-024-09575-8.

## Background

*Citrobacter* species are ubiquitous in the environment, and have long been considered pathogens of low virulence, causing infections less frequently compared to other Enterobacterales [1, 2]. As such, they are not considered classic nosocomial pathogens [3, 4]. In recent years, however, nosocomial *Citrobacter* spp. infections and hospital outbreaks have been increasingly reported [5, 6]. For instance, a *C. freundii* outbreak in a neonatal intensive care unit attracted public attention in Korea, after four neonates died of bacteraemia following receipt of a contaminated intravenous (IV) infusion [7].

Together with the growing body of evidence on *Citrobacter* spp. infections in hospitals, reports on antibiotic resistance among *Citrobacter* isolates have been also evolving, including reports on carbapenemase-producing [8], and AmpC β-lactamase (Amp-C) carrying isolates [9]. Several carbapenemases, carried on plasmids, have been described in *Citrobacter* spp., that can easily spread to other Enterobacterales species [10]. Nonetheless, the magnitude of *Citrobacter* spp. involvement as a clinically significant pathogen in hospitalized patients is not well established, and antibiotic resistance patterns in *Citrobacter* spp. have not been yet reviewed. Understanding the epidemiological features of this emerging pathogen, is essential to uncover its role in healthcare and to develop effective control strategies.

We conducted a systematic review and meta-analysis to evaluate the epidemiology of infections due to *Citrobacter* spp., and their antibiotic resistance patterns among hospitalized patients. We also examined the occurrence of hospital outbreaks due to *Citrobacter* spp.

## Methods

### Eligibility criteria

The eligibility criteria for study selection were defined using the PICOS framework (Patient, Intervention/exposure, Comparison, Outcome, Study design) [11]. Eligible study populations were hospitalized patients of any age, diagnosed with *Citrobacter* spp. infections, as well as those identified with colonization and/or infection due to *Citrobacter* during hospital outbreaks. Antibiotic resistance mechanisms of interest were 3rd generation cephalosporin and/or carbapenem resistance. Outcomes included prevalence and incidence of *Citrobacter* infections among hospitalized patients, prevalence/incidence of nosocomial *Citrobacter* infections and resistance percentages to the above-mentioned antibiotics. Frequency of reported hospital outbreaks due to *Citrobacter* was also evaluated. Eligible study designs were observational studies (cohort, cross-sectional, case–control studies, and case series), clinical trials, outbreak reports, and surveillance studies (Additional file 1, Review definitions). For an outbreak report to be included, *Citrobacter* spp. had to be the main implicated pathogen, defined as the responsible pathogen for at least one third of the detected cases. Eligible surveillance studies needed to be of at least one year duration and include a minimum of 30 *Citrobacter* isolates to be included. Studies reporting aggregate data on multiple Enterobacterales, or on other Enterobacterales, and studies focusing only on community-acquired infections were excluded.

### Information sources and search strategy

A detailed study protocol was published on 18 January 2023 on PROSPERO (CRD42023390084) [12]. Data sources were MEDLINE® (PubMed), Embase (Ovid), outbreak database [13], and grey literature including Global Index Medicus, US Centers for Disease Control and Prevention (CDC), and the European Centre for Disease Prevention and Control (ECDC) websites. The search included publications during the period Jan-2000 to Dec-2022 without language restriction. The Medline search strategy included a combination of MeSH terms and keywords, encompassing the following search concepts: *Citrobacter*, nosocomial (or healthcare- or hospital-acquired) infections, hospitalized patients, outbreak and surveillance. The search terms were modified as required for each of the other databases (Additional file 1, Search strategy). A systematic reference search was performed for all included *Citrobacter* outbreak studies.

### Study selection

A summary list of all titles/abstracts was generated according to the search terms. Searches from different databases were combined and de-duplicated using Covidence (Covidence systematic review software, Veritas Health Innovation, Melbourne, Australia) [14]. Single screening of titles/abstracts was performed by one reviewer (PF), complemented by additional discussion with a second reviewer (NHK), as needed. Two reviewers (PF and NHK) performed double full-text screening; any uncertainties were resolved by consensus. Data extraction was completed by a single reviewer (PF), with double extraction of 50% of included publications by a second reviewer (NHK). Data was extracted into dedicated forms designed in Covidence.

### Data extraction

The following data was extracted: bibliographic information, study design and setting, study characteristics (*i.e.*, objectives, sample size, age groups). Type(s) of clinical infections, and unit of analysis (infected patient or cultured isolate). Microbiological analysis methods (*i.e.* phenotypic and genotypic resistance evaluation) were also recorded.

Data on prevalence and incidence of *Citrobacter* spp. infections among hospitalized patients and resistance percentages of *Citrobacter* spp. isolates was retrieved. We recorded the percentage of isolates that were resistant to third generation cephalosporins or carbapenems or that produced extended-spectrum beta-lactamases (ESBL), AmpC beta-lactamases or carbapenemases. For outbreak study reports, data on setting, timing, and duration of each outbreak, detected outbreak source(s) and interventions implemented to halt the outbreak were collected.

### Methods of data synthesis

The characteristics of the included studies were described. Whenever available, prevalence and incidence rates were reported. Antimicrobial resistance percentages reported in observational and surveillance studies were meta-analysed to generate pooled estimates for both resistance mechanisms and resistance to specific antibiotic agents. Random effects models were used (when 3 or more studies reported specific resistance data). Freeman-Tukey double arcsine transformation was used to stabilize the variances [15], and statistical heterogeneity was assessed using the I^2^ statistic measure [16]. Studies focusing only on multidrug-resistant *Citrobacter* isolates and those reporting on < 10 *Citrobacter* isolates were excluded from the meta-analysis. Data on hospital outbreaks of *Citrobacter* spp. were summarized descriptively. Statistical analysis was done using ‘meta’ package, RStudio (Version 4.2.3).

### Risk of *bias* assessment

Risk of bias was assessed using the Joanna Briggs Institute (JBI) study design tools [17]. A study was defined at low risk of bias when it scored was ≥ 75% of the applicable score. Quality of reporting in hospital outbreaks was evaluated by compliance with the ORION recommendations [18]. One reviewer (PF) assessed risk of bias and reporting quality; risk of bias in 50% of all included studies was also evaluated by a second reviewer (NHK), with no major inconsistencies.

## Results

### Study selection and characteristics

**Fig. 1:**
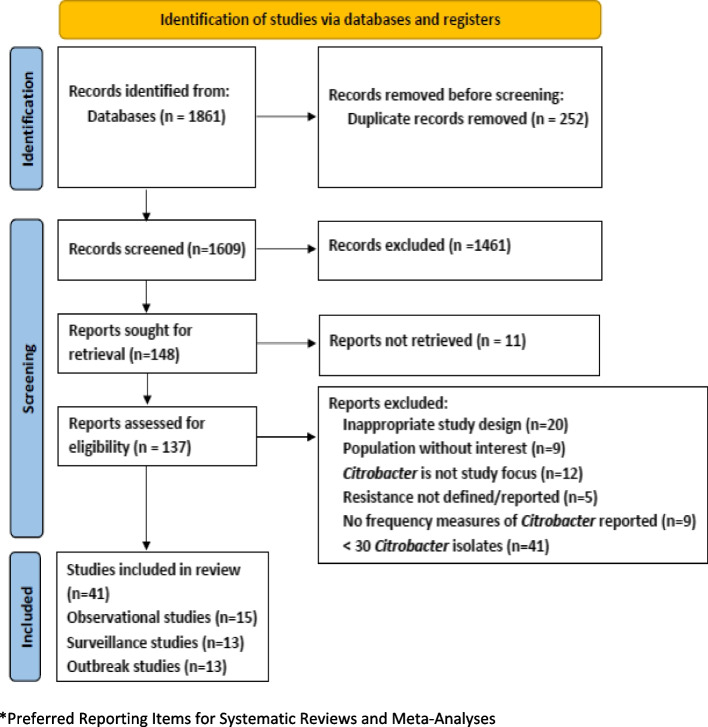
PRISMA* flowchart for the systematic review

A total of 1609 de-duplicated publications were identified and reviewed by title/abstract. Of these, 148 full-text articles were reviewed. Finally, 41 studies fulfilled the inclusion criteria (Fig. 1): 15 observational studies (10 cohort studies, four cross-sectional studies and one case-series); 13 surveillance studies and 13 outbreak reports. The main reasons for exclusion were surveillance studies including less than 30 *Citrobacter* spp. isolates (*n* = 41) and incompatible study design (*n* = 20). Most included observational studies were single-center studies (87%) whereas surveillance reports often included data from multi-center networks or reference laboratories (10/13, 77%; Table 1). Intensive care units (ICUs) were the most frequently implicated hospital department (14/28, 50%), with six studies focusing only on ICU patients (Table 1). Most observational studies were conducted in Asia, with the highest number of studies from India (*n* = 5). Three studies reported international surveillance data. Germany, Spain and USA were the countries contributing most *Citrobacter* surveillance data (three studies each).

**Table 1:** Characteristics of the included studies in the systematic review (*n* = 41)

Study ID (First author & year)	Country	Study design/ surveillance scope^a^	Number of sites	Hospital department(s)/type	Study period	Number of study participants or isolates – all pathogens	No. of patients with *Citrobacter* spp. / *Citrobacter* isolates	*Citrobacter* species
**Observational studies**								
**Adeyemo 2022 ** [19]	Nigeria	Cross-sectional	1	Hospital-wide	2016	359 p	83 p^e^	*Citrobacter* spp.
**Chao 2013** [20]	Taiwan	Cohort	1	NA	1990—2010	328 p	8 p	*C. freundii,* *C. koseri / diversus*
**Chen 2011** [21]	China	Cohort	1	NA	2005 – 2008	23 p	23 p^e^	*C. freundii*
**Gupta 2003 ** [22]	India	Cohort	1	ICU, pediatric and surgical	1998 – 2001	48 p	48 p^e^	*C. freundii;* *C. koseri / diversus*
**Kim 2003** [23]	Korea	Cohort	1	NA	1991 – 2000	105 p	105 p^e^	*C. freundii*
**Lavigne 2007** [24]	France	Cohort	1	Hospital-wide	2003 –2004	45 p	45 p^e^	*C. freundii; C. braakii;* *C. koseri / diversus*
**Lee 2019** [2]	Korea	Cohort	1	Hospital-wide, hematology and neurosurgery	2007 –2017	43 p	43 p^e^	*C. freundii, C. braakii,* *C. koseri / diversus,* *C. amalonaticus, C. youngae*
**Liu 2007** [25]	Taiwan	Cases-series	1	ICU	2002 – 2003	12 p	12 p^e^	*C. freundii*
**Lodise 2017** [26]	USA	Cohort	178	Hospital-wide and ICU	2011—2014	60,551 p 94,851 i	2827 p 3043 i	*Citrobacter* spp.
**Metri 2011** [27]	India	Cross-sectional	1	ICU, NICU, surgical, pediatric, urology, OBG and medical department	2007- 2011	563 p	563 p^e^	*C. freundii;* *C. koseri / diversus*
**Mirzaei 2021** [28]	Iran	Cross-sectional	2	Pediatric	2017 – 2019	295 i	65 i	*C. freundii*
**Mishra 2016** [29]	India	Cohort	1	ICU, NICU, burn ICU and pediatric ICU	2013 –2014	510 p	41 p^e^	*C. freundii*
**Mohanty 2007** [30]	India	Cohort	1	ICU and hospital-wide	2004	205 p	205 p^e^	*C. freundii,* *C. koseri / diversus*
**Norouzi Bazgir 2020** [31]	Iran	Cross-sectional	1	ICU, burn unit and outpatient	2016 -2017	109 i	109 i	*C. freundii*
**Praharaj 2016** [32]	India	Cohort	1	Hospital-wide and surgical ICU	2010 – 2013	221 i	221 i	*C. freundii;* *C. koseri / diversus*
**Surveillance studies**								
**Arana 2017** [33]	Spain	National	115	NA	2013 – 2015	4129 i	119 i^e^	*C. freundii; C. braakii; C. koseri /diversus;* *C. amalonaticus*
**Goossens 2005** [34]	Multi-country^**b**^	International	41	ICU, medical units	1997 – 2004	23,929 i	1333 i	*Citrobacter* spp.
**Hawser 2010** [35]	Multi-area/country^**c**^	International	144	ICU, medical, surgical and emergency	2002 – 2007	32,113 i	973 i	*C. freundii*
**Ishii 2006** [36]	Japan	National	100	NA	2004	9347 i	834 i	*C. freundii*
**Jones 2003** [37]	Multi-country^d^	Regional	570	ICU and hospital-wide	2001	85,098 i	1148 i	*Citrobacter* spp.
**Logan 2015** [38]	USA	National	300	ICU and hospital-wide, pediatric and outpatient	1999 – 2012	316253 i	6730 i	*C. freundii;* *C. koseri /diversus*
**Mylvaganam 2017** [39]	Norway	Sub-regional	2	NA	2006 – 2013	73,440 i	1139 i	*C. freundii, C. braakii;* *C. koseri /diversus*
**Nishio 2004** [40]	Japan	National	13	ICU, NICU, pediatric and internal medicine	2000 –2002	19,753 i	544 i	*C. freundii*
**Orrett 2000** [41]	Trinidad	Hospital	1	NA	1997	1129 i	31 i	*Citrobacter* spp.
**Raisanen 2021** [42]	Finland	Hospital	8	NA	2016 – 2020	20 p	20 p^e^	*C. freundii*
**Rezaei 2016** [6]	Iran	Hospital	1	ICU, medical and surgical	2013	50 p	50 p^e^	*C. freundii*
**Shetty 2007** [43]	India	Hospital	1	NA	2002–2004	709 i	709 i	*Citrobacter* spp.
**Yao 2021** [3]	Germany	National	61	NA	2017 – 2019	512 i	52 i^e^	*C. freundii; C. braakii;* *C. koseri /diversus;* *C. portucalensis,* *C. europaeus*
**Outbreak studies**								
**De Geyter 2017** [44]	Belgium	-	1	Tertiary care	2015	21 p	5 p	*C. freundii*
**Entezari 2016** [45]	Iran	-	2	Ophthalmologic hospital	2015	21 p	13 p	*Citrobacter* spp.
**Gaibani 2013** [46]	Italy	-	1	NA	2012	8 p	8 p	*C. freundii*
**Gobeille Paré 2020** [47]	Canada	-	4	Tertiary care	2016—2018	65 p	63 p	*C. freundii*
**Jimenez 2017** [48]	USA	-	1	Tertiary care	2014 -2015	6 p	6 p	*C. freundii*
**Jolivet 2021** [49]	France	-	1	NA	2016—2019	37 p	22 p	*C. freundii*
**Muta 2006** [50]	Japan	-	1	NA	NA	31 p	4 p	*C. koseri / C. diversus*
**Nada 2004** [51]	Japan	-	1	NA	2022	7 p	7 p	*C. freundii*
**Pletz 2018** [52]	Germany	-	1	Tertiary care	2016	76 p	76 p	*C. freundii*
**Rodel 2019** [53]	Germany	-	1	Tertiary care	2016–2017	56 i	23 i	*C. freundii*
**Royer 2020** [54]	France	-	1	Rehabilitation center	2019	5 p	5 p	*C. amalonaticus*
**Schweizer 2019** [55]	Germany	-	2	NA	2016	7 p	7 p	*C. freundii*
**Segal 2022** [56]	Israel	-	1	NA	2020	7 p	2 p	*C. freundii*

*ESBL* Extended Spectrum Beta-Lactamase, *CPC* Carbapenemase-producing *Citrobacter* spp., *ICU* Intensive care unit, *IAI* Intra-abdominal infections, *NICU* Neonatal Intensive care unit, *OBG* Obstetrics and Gynaecology, *p* patients, *i* isolates

^a^Study design in observational studies; surveillance scope for surveillance studies

^b^Germany, Greece, USA, UK, Spain, Belgium, Croatia, Czech Republic, Finland, Poland, Russia and Turkey

^c^Asia Pacific, Europe, Latin America, Middle East, Africa and North America

^d^France, Germany, Italy, USA and Spain

^e^Observational and surveillance studies reporting one *Citrobacter* isolate per patient (number of isolates = number of patients); NA: not reported/specified

### *Citrobacter* infections among hospitalized patients

Out of 28 observational and surveillance studies, 15 studies (54%) focused on patients infected with *Citrobacter*, while the remaining included also other Enterobacterales infections. Across all studies, *C. freundii* was the most frequently species (reported in 22/28, 79%), followed by *C. koseri/C. diversus* (11/28, 39%) and *C. braakii* (5/28, 18%). Other species included *C. amalonaticus, C. youngae, C. portucalensis and C. europaeus* (Table 1). Most studies provided a clear definition of clinical infection; yet 13/28 studies (46%) only reported on *Citrobacter* spp. growth in clinical cultures without providing additional clinical information (Table 2). *Citrobacter* bloodstream infections (BSI) were the focus of four studies [2, 5, 22, 23].

**Table 2:** Summary of epidemiological and microbiological features in the included observational and surveillance studies

Study ID	Country	Resistance evaluation	Phenotypic and genotypic resistance mechanism checked	Resistance encoding-genes or mechanisms identified	Others resistance genes or mechanisms identified	Infection definition: clinical diagnosis vs culture	Type of infections related to *Citrobacter* spp.
Observational studies							
Adeyemo 2022	Nigeria	Phenotypic; genotypic	CR, 3GC, ESBL/Ampc-producing	ESBL-producing	NA	Clinical	UTI, RTI, IAI, SSTI, SSI and Sepsis
Chao 2013	Taiwan	Phenotypic	CR, 3GC-R	NA	NA	Clinical	Sepsis
Chen 2011^a^	China	Phenotypic, genotypic	CR, Carbapenemase-producing, 3GC-R, ESBL-producing, AmpC	KPC, KPC-2, IMP-1, IMP-2, OXA-69, OXA-23, OXA-58, OXA-51 and CTX-M-14	AmpC-producing (CMY-2)	Clinical	UTI
Gupta 2003	India	Phenotypic	3GC-R	NA	NA	Clinical	BSI
Kim 2003^a^	Korea	Phenotypic	CR, 3GC-R	NA	NA	Clinical	BSI, UTI, RTI, biliary infections
Lavigne 2007^a^	France	Phenotypic; genotypic	3GC-R, ESBL-producing	TEM-3	AmpC-producing	Clinical	UTI
Lee 2019^a^	Korea	Phenotypic	CR, 3GC-R	NA	NA	Clinical	BSI, UTI, SSTI, CLABSI and gastrointestinal infections
Liu 2007^a^	Taiwan	Phenotypic; genotypic	CR, 3GC-R, ESBL-producing, AmpC	TEM-1, SHV-12 and CTX-M-27	AmpC-producing	Clinical	BSI
Lodise 2017	USA	Phenotypic	CR	NA	NA	Clinical	BSI, UTI and IAI
Metri 2011^a^	India	Phenotypic	CR, 3GC-R	NA	NA	Clinical	BSI, UTI and wound infections
Mirzaei 2021	Iran	Phenotypic	CR, 3GC-R, rpoB gene	NA	NA	Culture	-
Mishra 2016	India	Phenotypic	3GC-R and MDR	NA	NA	Culture	UTI
Mohanty 2007^a^	India	Phenotypic	CR, 3GC-R	NA	NA	Clinical	BSI, UTI, RTI and SSTI
Norouzi Bazgir 2020	Iran	Phenotypic	CR, 3GC-R, MDR	NA	NA	Culture	BSI
Praharaj 2016	India	Phenotypic; genotypic	CR, Carbapenemase-producing, 3GC-R, ESBL/AmpC-producing; MBL	VIM-2, NDM-1, TEM-1, SHV-12, SHV-18 and CTX-M-15	AmpC-producing	Clinical	BSI, UTI, RTI, SSTI, IAS and miscellaneous
Surveillance studies							
Arana 2017	Spain	Phenotypic, genotypic	CR, Carbapenemase-producing, MDR	NDM-1, OXA-48, VIM-2, KPC-2, SHV-12, CTX-M-9 and CTX-M-15	-	Culture	BSI, UTI and wound infections
Goossens 2005		Phenotypic, genotypic	CR, Carbapenemase-producing, 3GC-R, ESBL/AmpC-producing, MDR	NA	AmpC-producing	Culture	NA
Hawser 2010	Asia Pacific, Europe, Latin America, Middle East, Africa and North America	Phenotypic	CR, 3GC-R, ESBL-producing	NA	-	Clinical	IAI
Ishii 2006	Japan	Phenotypic	CR, 3GC-R		-	Culture	BSI, UTI, RTI and IAI
Jones 2003		Phenotypic	CR, 3GC-R, MDR	NA	-	Clinical	SSTI
Logan 2015	USA	Phenotypic	CR, Carbapenemase-producing, MDR	NA	-	Culture	NA
Mylvaganam 2017	Norway	Phenotypic	CR, 3GC-R, ESBL/AmpC-producing	NA	AmpC-producing	Culture	BSI and UTI
Nishio 2004	Japan	Phenotypic, genotypic	CR, 3GC-R, MDR	IMP-1	-	Culture	NA
Orrett 2000	Trinidad	Phenotypic	CR, 3GC-R	-	-	Culture	UTI and wound infections
Raisanen 2021	Finland	Phenotypic, genotypic	CR, ESBL/AmpC-producing	KPC-2, KPC-3, OXA-181, GES-5 and CTX-M-15	-	Culture	BSI, UTI and wound infections
Rezaei 2016	Iran	Phenotypic, genotypic	CR, 3GC-R, ESBL/Ampc-producing	ESBL-producing	-	Culture	NA
Shetty 2007	India	Phenotypic	CR, 3GC-R, MDR	NA	-	Culture	BSI, UTI, RTI and wound infections
Yao 2021	Germany	Phenotypic, genotypic	CR, Carbapenemase-producing, MDR	KPC-2, KPC-3, NDM-5, NDM-1, VIM-2, VIM-4, OXA-48, OXA-162, CTX-M-1, CTX-M-3, CTX-M-9, CTX-M-15, TEM-1, OXA-1, OXA-17 and OXA-162	strA, strB, qnrA1, qnrB, qnrS, dfrA, sul1, sul2, tet(B), tet(A), aac(3)-IIa, aac(6)-If, aadA1, catB, mph(A), mph(E), msr(E), ARR-3,CMY	Clinical	BSI, UTI and wound infections

*BSI* Bloodstream Infections, *UTI* Urinary-tract Infections, *RTI* Respiratory-tract Infections, *IAI* Intra-abdominal Infections, *CR* Carbapenem-resistance, 3GC-R Third-Generation Cephalosporins resistance, MDR Multi-drug resistant, *ESBL* Extended Spectrum Beta-lactamase, *CLABSI* Catheter-line associated to bloodstream infections, *SSI* Surgical Site Infection, *IAI* Intra-abdominal Infection, *SSTI* Skin and Soft Tissue Infection

^a^Studies reporting separately on *Citrobacter* nosocomial infections

In observational studies, a median of 65 patients with *Citrobacter* infections were included per study (interquartile range (IQR), 42–157), contributing to a total of 4617 *Citrobacter* patients. In surveillance studies, a median of 279 *Citrobacter* isolates were included per study (IQR, 52–834), contributing to a total of 6582 isolates. An increasing number of patients with *Citrobacter* infection/colonization were reported in observational studies after 2010 (Additional file 1, Figure S1).

Data scarcity prevented generating pooled incidence estimates; two studies provided denominator data quantifying the size of population at risk, yielding a cumulative incidence of 0.175 and 0.035 episodes per 1000 patients for *Citrobacter* BSI and invasive *Citrobacter* infections, respectively [23, 26].

Among hospitalized patients, UTI was the most frequently reported *Citrobacter* infection (17/28 studies, 61%) followed by BSI (15/28 studies, 54%), and respiratory-tract infection (RTI) in 8 studies (29%, Table 2). In most studies the exact date of infection-onset was not clearly defined. Yet, seven studies reported separately on patients with hospital-acquired *Citrobacter* infections; 85% (838/990) of hospitalized *Citrobacter* patients in these studies had a nosocomial infection. In three studies reporting patient mortality after nosocomial *Citrobacter* BSI, a case fatality ratio of 34% (36/106 patients) was found [2, 23, 25].

### *Citrobacter* antibiotic resistance patterns among hospitalized patients

A total of 11,199 *Citrobacter* isolates were analyzed (4617 and 6582 from observational and surveillance studies, respectively). Urine and blood isolates were most common in observational studies, whereas the specimen type was often unspecified in surveillance studies (Additional file 1, Figure S2).

Phenotypic resistance to antibiotics was assessed in all included studies, and genotypic resistance in 11/28 studies (Table 2). Pooled resistance percentages from observational studies were higher than those from surveillance studies (Table 3). The pooled percentage of ESBL-producing *Citrobacter* was 22.2% (95% CI 3.5% – 50.3%, 8 studies), and for AmpC production, 33.3% (95% CI 13.2% – 53.4%, 4 studies, Table 3). Pooled resistance percentages for specific antibiotic agents in observational studies ranged between 26.4% for imipenem resistance (95%CI 0.0% – 54.6%, 6 studies) and 64.9% for ceftazidime resistance (95%CI 44.5%—82.9%, 6 studies), and in surveillance studies, between 0.1% for imipenem resistance (95%CI 0.0%-0.4%, 5 studies) and 21.7% for ceftazidime resistance (95%CI 5.0% – 45.4%, 6 studies). Of note, high resistance percentages were observed for other antibiotic agents, such as ciprofloxacin and gentamicin. Forest plots for resistance analyses are provided in Additional file 2.

**Table 3:** Pooled estimates of resistance percentage of included *Citrobacter* spp. isolates, per resistance mechanism and antibiotic agent stratified by study category

**Resistance pattern**	**Observational studies**					**Surveillance studies**					**Overall**				
	**Number of studies**	**Total number of *Citrobacter* isolates**	**Pooled resistance percentage**	**95% CI**	**I**^**2**^	**Number of studies**	**Total number of *Citrobacter* isolates**	**Pooled resistance percentage**	**95% CI**	**I**^**2**^	**Number of studies**	**Total number of *Citrobacter* isolates**	**Pooled resistance percentage**	**95% CI**	**I**^**2**^
**Resistance mechanism**															
**ESBL-producing**	3	349	**44.2**	9.9 – 82	98%	4	458	**6.1**	0.0 – 22.9	93%	8	807	**22.2**	3.5 – 50.3	99%
**Carbapenemase-producing**	-	-	**-**	-	-	2	-	**-**	-	-	2	-	**-**	-	-
**AmpC-producing**	2	266	**49.1**	29 – 69.1	85%	2	377	**18.3**	3.2 – 33.4	90%	4	643	**33.3**	13.2–53.5	98%
**Phenotypic resistance**															
**Imipenem**	7	1098	**22.6**	0.0 – 47.6	99%	5	3260	**0.1**	0.0 – 0.4	46%	12	4358	**13.2**	0.0 – 28.5	98%
**Meropenem**	4	391	**33.0**	1.5 – 77.6	99%	3	1038	**2.0**	0.0 – 8.4	90%	7	1429	**16.0**	0.5 – 44.2	99%
**Ceftazidime**	6	1025	**64.9**	44.5 – 82.9	94%	6	3241	**21.7**	5.0 – 45.4	99%	12	4266	**42.4**	23.3 – 62.7	99%
**Cefotaxime**	5	409	**56.6**	35.1 – 76.8	95%	3	1497	**39.7**	15.1 – 67.4	99%	8	1906	**49.8**	33.1 – 66.6	99%
**Piperacillin/ tazobactam**	4	851	**27.4**	17.2 – 38.8	96%	5	2380	**29.2**	14.0 – 47.4	98%	9	3231	**27.4**	17.2 – 38.8	98%
**Amikacin**	8	1171	**33.3**	19.6 – 47.1	98%	3	1962	**16.9**	0.0 – 41.1	100%	11	3133	**28.7**	16.5– 40.9	99%
**Gentamicin**	7	973	**55.4**	32.3 – 78.3	97%	4	1990	**21.5**	1.4 – 54.1	100%	11	2963	**42.5**	22.5 – 64.0	99%
**Ciprofloxacin**	7	1137	**52.9**	30.8 – 74.5	97%	4	2439	**25.0**	8.3 – 46.9	99%	11	3576	**42.1**	25.0 – 60.2	99%
**Co-trimoxazole**	6	923	**55.6**	23.6 – 85.3	99%	3	1666	**16.2**	0.0 – 52.7	99%	9	2589	**41.6**	18.8– 69.1	99%

The mixed specimen isolates included were obtained from rectal swabs and various clinical specimens such as urine, blood, pus, respiratory secretions (sputum, endotracheal secretions, broncho-alveolar lavage (BAL) and bronchial wash) and others sterile body fluids

Large heterogeneity was observed in the meta-analysis for all antibiotics. Significant subgroup differences between observational and surveillance studies were found for imipenem and ceftazidime (Additional file 2). In a subgroup analysis of observational studies focusing only on *Citrobacter* BSI, pooled resistance percentages to cefotaxime of 46.5% (95%CI 32.6–60.6, I^2^ = 71%, 4 studies) and negligible resistance to imipenem (95% CI 0 – 0.6, I^2^ = 0%, 3 studies) were found.

### Nosocomial *Citrobacter* Outbreaks

Thirteen *Citrobacter* hospital outbreak reports were included, with a notable increase in reporting after 2016 (Table 4). Outbreaks frequently occurred in ICUs (*n* = 5), surgery and hematology units (3 each). *C. freundi* was the most often implicated species (10/13). Frequently detected carbapenemase and ESBL-production genes in outbreak isolates were OXA-48, KPC, CTX-M and AmpC cephalosporinase genes (Table 4). Two point-source outbreaks were identified, one tracked back to a staff member and the other to use of a contaminated solution for intravitreal injection [45, 56]. Other outbreaks were attributed to the hospital kitchen, or hospital toilets and sinks (Table 4). Non-point-source outbreaks lasted for a median duration of 212 days (IQR, 134–471), and a median of seven patients with *Citrobacter* infection and/or colonization were detected per outbreak (IQR, 5–16). The case fatality of *Citrobacter* infection was 7% (6/89 patients) based on three outbreak studies reporting mortality [48, 52, 55]. *Citrobacter* outbreaks were reported as definitively controlled following the implementation of various preventive measures in 7/13 reports (Additional file 1, Table S1).

**Table 4:** Main characteristics of included outbreak reports

Study id	Country	Outbreak period	Outbreak duration (days)	Type of department/ medical units	Number of *Citrobacter* cases	Type of infection	Resistance mechanism	Resistance encoding genes identified	Other resistance encoding genes identified	Outbreak source	Numberof deaths
Entezari 2016^c^	Iran	2015	3^**a**^	NA	13	Endophthalmitis	NA	NA	**-**	**Contaminated intravitreal injection**	NA
De Geyter 2017^c^	Belgium	2015	365	ICU	5	RTI and IAI	CPC	OXA-48 and NDM	**-**	**Sinks**	NA
Gaibani 2013^c^	Italy	2012	15	NA	8	NA	CPC	VIM-1 and NDM-1	-	NA	NA
Gobeille Pare 2020^d^	Canada	2016 -18	1006	ICU and nephrology	63	UTI, RTI	CPC	OXA-204 and OXA-48	-	NA	NA
Jimenez 2017^c^	USA	2014 -15	212	Surgical, neurology/ orthopedics	6	UTI, RTI and IAI	CPC and ESBL-producing	KPC-3, CMY-49, CMY-70, CMY-65, TEM-1A, OXA-2, OXA-9, and TEM-1B	qnrB34, qnrB19, qnrB38, sul1, sul2, tet(D), dfrA, dfrA8, dfrA12, aadB, strA/strB, aac(6’)Ib-cr, aadA1 and aadA7	NA	**0**
Jolivet 2021^c^	France	2016 -19	1263	Hematology	22	BSI, UTI, RTI	CPC	OXA-48	**-**	**Toilets**	NA
Muta 2006^c^	Japan	Unspecified	540	Hematology	4	Sepsis, gastroenteritis	ESBL	CTX-M-2	-	NA	NA
Nada 2004^d^	Japan	2022	123	Surgical	7	Biliary and wound infections	ESBL	AmpC-cephalosporinase	-	NA	NA
Pletz 2018^d^	Germany	2016	144	ICU, hematology, surgical, oncology, dermatology, and neonatology	76	BSI	CPC	VIM	aadA	**Foodborne**^**b**^	**3**
Rodel 2019	Germany	2016–17	402	Hospital-wide	23	BSI	CPC	VIM	**-**	**Foodborne**^**b**^	NA
Royer 2020^d^	France	2019	17	NA	5	NA	CPC and ESBL-producing	NDM-1, TEM-1, SHV-12, OXA-1, CMY-4, TEM-1B	mcr-9, arr-3, aac(6')-Ib-cr, qnrB32, dfrA14, sul1, sul2, dfrA14, catB3, floR, tet(A), aph(3'')-Ib, aph(3')-VI, aph(6)-Id	NA	NA
Segal 2022	Israel	2020	2^a^	ICU	2	BSI	NA	NA	**-**	**Healthcare worker**	NA
Schweizer 2019^c^	Germany	2016	180	ICU	7	RTI	CPC and ESBL-producing	KPC-2, OXA-1, TEM-1B	aac (6’)-Ib-cr, OXY-like and qnrB2	NA	**3**

*ESBL* Extended Spectrum Beta-Lactamase, *CPC* Carbapenemase-producing *Citrobacter* spp*.*, *p* patients, *i* isolates

^a^point-source outbreak

^b^Hospital kitchen foodborne (including prepared vegetable salads, puddings and mixing machine)

^**c**^Outbreak reports in which *Citrobacter* spread was controlled

^d^Outbreak reports in which *Citrobacter* spread was not contained

### Risk of *bias* and quality of reporting assessment

High risk of bias was observed (6/10 cohort studies and 2/4 cross-sectional studies, additional file 1, Figures S4-S7). Domains of high risk of bias were confounder identification and adjustment, exposure classification and adequacy of follow-up. Conversely, a good quality of outbreak reporting was found as evaluated by the ORION statement.

## Discussion

To the best of our knowledge, this is the first systematic review focusing on *Citrobacter* spp. infections among hospitalized patients. By including 41 studies across different study designs, we could portray a comprehensive picture of endemic and epidemic *Citrobacter* spp. infections in the hospital setting. *C. freundii* was found as an important, emerging multidrug-resistant pathogen, causing diverse nosocomial infections and outbreaks, increasingly reported since 2016. Interestingly, half of all included studies (21/41) were conducted in Asian countries, hinting at the importance of *Citrobacter* as a multidrug-resistant pathogen in that region.

Our findings confirm that *Citrobacter* spp. frequently harbour multiple resistance elements; several types of carbapenemase, beta-lactamase and AmpC-cephalosporinase resistance genes were found in the included studies. Overall, high antibiotic resistance percentages were identified in *Citrobacter* isolates, especially for 3rd generation cephalosporins, gentamicin and fluoroquinolones. This is an alarming finding, limiting the available treatment options for *Citrobacter* infections [4, 57]. Of note, pooled resistance percentages were lower among isolates collected for surveillance purposes compared to those in observational studies, a finding that can be explained by the different target populations in these types of studies [58].

We found substantial resistance to cefotaxime in *Citrobacter* blood isolates (46.5%), which is comparable to cefotaxime resistance in other Enterobacterales monitored in the Global Antimicrobial Resistance Surveillance System (GLASS) network, with 63% of *Klebsiella pneumoniae* and 38.5% of *Escherichia coli* found resistant to cefotaxime in blood isolates collected in 2020 [59]. In light of this finding, systematic monitoring of antimicrobial resistance in *Citrobacter* spp. should be considered.

Although resistance percentages are important for the clinician prescribing an empirical therapy, these are less informative for public-health purposes; they are often based on biased estimates, and do not reflect the magnitude of the problem as rate-based estimates [60]. Due to data scarcity, we were unable to generate pooled estimates of the incidence of multidrug-resistant *Citrobacter* infections.

Many *Citrobacter* hospital outbreaks identified in our review were related to the hospital environment (sinks, toilets, and kitchens); this finding aligns with the study by Hamerlinck et al., who showed that carbapenem-resistant *Citrobacter* can evolve in the hospital aquatic environment, and suggested long-term persistence of this pathogen in the hospital plumbing system [61]. Of note, *Citrobacter* was also responsible for two point-source outbreaks, emphasizing its ability to contaminate a common source. Transition from epidemic to endemic occurrence was observed in almost one third of included outbreaks, for which definitive outbreak control was not achieved according to the publication, despite multiple interventions. The diverse outbreak sources and transmission patterns of *Citrobacter* call for increased awareness of the risk of nosocomial *Citrobacter* clusters and reinforcement of infection control measures related to aseptic procedures, pharmaceutical preparations and environmental hygiene.

*Citrobacter* infections may cause life-threatening infections [24, 62]. In a historical cohort study from Taiwan*,* 45 patients with *Citrobacter* BSI had an overall case-fatality ratio of 33% [63], similar to the ratio of 34% found in our review. Moreover, we documented a case-fatality ratio of 7% among patients affected by *Citrobacter* outbreaks.

Large heterogeneity was observed in the pooled resistance estimates that could be related to true differences in epidemiologic or microbiological methods, or patient case-mix. We tried to control for heterogeneity due to study design/case-mix by analysing resistance percentages in observational and surveillance studies separately. A subgroup analysis of resistance percentages in blood isolates was also conducted. However, the number of studies identified did not allow for further subgroup analyses.

This systematic review has limitations. First, our findings might underestimate resistant *Citrobacter* involvement in surveillance studies and hospital outbreaks, as we excluded surveillance studies with less than 30 *Citrobacter* isolates and outbreaks in which *Citrobacter* spp. was not the main pathogen. Second, we aimed to assess the magnitude of hospital-acquired *Citrobacter* infections; however, only seven studies clearly distinguished between community vs. hospital-acquisition. Nonetheless, 85% of *Citrobacter* infections were hospital-acquired when reported. Third, there was large variability in microbiologic methods, which might have affected the results of the individual studies. Forth, multiple specimen types were included and stratified analysis was only possible for blood isolates. Last, publication bias might have affected our findings both for resistance percentages and involvement of *Citrobacter* spp. in hospital outbreaks.

## Conclusions

In conclusion, based on the reviewed studies, *Citrobacter* represents an emerging multidrug-resistant pathogen in hospitalized patients. The increased resistance among *Citrobacter* isolates, its ability to harbor numerous resistance genes, and its active role in hospital outbreaks all make *Citrobacter* an important, global patient safety risk. Our findings call for inclusion of *Citrobacter* spp. in surveillance networks as a pathogen of epidemiological significance, as done for *Enterobacter* spp. In addition, future studies need to address the role of *Citrobacter* spp. in nosocomial infections and better elucidate its reservoirs and transmission routes in the hospital environment.

### Supplementary Information

Supplementary Material 1.

Supplementary Material 2.

## Data Availability

The datasets used and/or analyzed during the current study are available from the corresponding author based on reasonable request.

## Abbreviations

3GC-R: Third Generation cephalosporins resistance
AmpC: Amplified Cephalosporinase
BSI: Bloodstream infections
CR: Carbapenem-resistance
CI: Confidence Interval
CLSI: Clinical Laboratory Standard Institute
ESBL: Extended-spectrum beta-lactamase
IAI: Intra-abdominal infections
ICU: Intensive care unit
IV: Intravenous
IQR: Interquartile range
MDR: Multi-drug resistant
NICU: Neonatal intensive care unit
OBG: Obstetrics and Gynecology
RTI: Respiratory-tract infections
UTI: Urinary-tract infections

## References

[1] Pepperell C, Kus JV, Gardam MA, Humar A, Burrows LL (2002). Low-Virulence Citrobacter Species Encode Resistance to Multiple Antimicrobials. Antimicrob Agents Chemother.

[2] Lee R, Choi S-M, Jo SJ, Lee J, Cho S-Y, Kim S-H (2019). Clinical Characteristics and Antimicrobial Susceptibility Trends in Citrobacter Bacteremia: An 11-Year Single-Center Experience. Infect Chemother.

[3] Yao Y, Falgenhauer L, Falgenhauer J, Hauri AM, Heinmüller P, Domann E (2021). Carbapenem-Resistant Citrobacter spp. as an Emerging Concern in the Hospital-Setting: Results From a Genome-Based Regional Surveillance Study. Front Cell Infect Microbiol..

[4] Ranjan KP, Ranjan N (2013). Citrobacter: An emerging health care associated urinary pathogen. Urol Ann.

[5] Liu L, Qin L, Hao S, Lan R, Xu B, Guo Y (2020). Lineage, Antimicrobial Resistance and Virulence of Citrobacter spp. Pathogens.

[6] Rezaei M, Akya A, Elahi A, Ghadiri K, Jafari S (2016). The clonal relationship among the Citrobacter freundii isolated from the main hospital in Kermanshah, west of Iran. Iran J Microbiol.

[7] Bae JY, Kang CK, Choi SJ, Lee E, Choe PG, Park WB (2018). Sudden Deaths of Neonates Receiving Intravenous Infusion of Lipid Emulsion Contaminated with *Citrobacter freundii*. J Korean Med Sci.

[8] Krankenhaushygiene und Infektionsprävention (KRINKO) Robert Koch-Institut (RKI), Empfehlung der Kommission für zu Hygienemaßnahmen bei Infektionen oder Besiedlung mit multiresistenten gramnegativen Stäbchen. Bundesgesundheitsblatt. 2012;55:1311–54. 10.1007/s00103-012-1549-5 23011096

[9] Iredell J, Brown J, Tagg K (2016). Antibiotic resistance in Enterobacteriaceae: Mechanisms and clinical implications. BMJ.

[10] Ledda A, Cummins M, Shaw LP, Jauneikaite E, Cole K, Lasalle F, et al. Hospital outbreak of carbapenem-resistant Enterobacterales associated with a bla OXA-48 plasmid carried mostly by Escherichia coli ST399. Microb Genomics. 2022;8(4):000675. 10.1099/mgen.0.000675 PMC9453065 35442183

[11] Methley AM, Campbell S, Chew-Graham C, McNally R, Cheraghi-Sohi S (2014). PICO, PICOS and SPIDER: a comparison study of specificity and sensitivity in three search tools for qualitative systematic reviews. BMC Health Serv Res.

[12] Fonton P, de Kraker M, Abbas M, Hassoun-Kheir N, Harbarth S. The epidemiology of nosocomial Citrobacter spp: A systematic review. PROSPERO 2023 CRD42023390084. https://www.crd.york.ac.uk/prospero/display_record.php?ID=CRD42023390084. Accessed 30 Sep 2023.

[13] Outbreak Database - Home. https://www.outbreak-database.com/Home.aspx. Accessed 6 Oct 2023.

[14] Covidence - Better systematic review management. https://www.covidence.org/. Accessed 16 Feb 2024.

[15] Doi SA, Xu C (2021). The Freeman-Tukey double arcsine transformation for the meta-analysis of proportions: Recent criticisms were seriously misleading. J Evid-Based Med.

[16] Higgins JP, Thomas J, Chandler J, Cumpston M, Li T, Page MJ, Welch VA, editor(s). Cochrane Handbook for Systematic Reviews of Interventions Version 6.4 (updated August 2023). Cochrane, 2023. Available from training.cochrane.org/handbook.

[17] JBI Critical Appraisal Tools | JBI. https://jbi.global/critical-appraisal-tools. Accessed 24 Oct 2023.

[18] Stone SP, Cooper B, Kibbler CC, Cookson BD, Roberts JA, Medley G (2007). The ORION statement: guidelines for transparent reporting of Outbreak Reports and Intervention studies Of Nosocomial infection. J Antimicrob Chemother.

[19] Adeyemo AT, Odetoyin BW, Onipede AO (2022). Prevalence and risk factors for extended-spectrum β-lactamase_producing Gram-negative bacterial infections in hospitalized patients at a tertiary care hospital, southwest Nigeria. Afr J Clin Exp Microbiol.

[20] Chao C-T, Lee S-Y, Yang W-S, Chen H-W, Fang C-C, Yen C-J (2013). Citrobacter Peritoneal Dialysis Peritonitis: Rare Occurrence with Poor Outcomes. Int J Med Sci.

[21] Chen Y-S, Wong W-W, Fung C-P, Yu K-W, Liu C-Y (2002). Clinical features and antimicrobial susceptibility trends in Citrobacter freundii bacteremia. J Microbiol Immunol Infect Wei Mian Yu Gan Ran Za Zhi.

[22] Gupta R, Rauf SJ, Singh S, Smith J, Agraharkar ML (2003). Sepsis in a renal transplant recipient due to Citrobacter braakii. South Med J.

[23] Kim BN, Woo JH, Ryu J, Kim YS (2003). Resistance to extended-spectrum cephalosporins and mortality in patients with Citrobacter freundii bacteremia. Infection.

[24] Lavigne J-P, Defez C, Bouziges N, Mahamat A, Sotto A (2007). Clinical and molecular epidemiology of multidrug-resistant Citrobacter spp. infections in a French university hospital. Eur J Clin Microbiol Infect Dis..

[25] Liu C-P, Weng L-C, Tseng H-K, Wang N-Y, Lee C-M (2007). Cefotaxime-resistant Citrobacter freundii in isolates from blood in a tertiary teaching hospital in Northern Taiwan. J Infect.

[26] Lodise T, Ye MJ, Zhao Q (2017). Prevalence of Invasive Infections Due to Carbapenem-Resistant Enterobacteriaceae among Adult Patients in U.S. Hospitals. Antimicrob Agents Chemother..

[27] Metri BC, Jyothi P, Peerapur BV (2011). Anti-microbial resistance profile of Citrobacter species in a tertiary care hospital of Southern India. Indian J Med Sci.

[28] Mirzaei B, Babaei R, Bazgir ZN, Goli HR, Keshavarzi S, Amiri E (2021). Prevalence of Enterobacteriaceae spp. and its multidrug-resistant rates in clinical isolates: A two-center cross-sectional study. Mol Biol Rep..

[29] Mishra MP, Sarangi R, Padhy RN (2016). Prevalence of multidrug resistant uropathogenic bacteria in pediatric patients of a tertiary care hospital in eastern India. J Infect Public Health.

[30] Mohanty S, Singhal R, Sood S, Dhawan B, Kapil A, Das BK (2007). Citrobacter infections in a tertiary care hospital in Northern India. J Infect.

[31] Norouzi Bazgir Z, Mirzaei B, Haghshenas MR, Goli HR, Shafaie E (2020). Multi-drug Resistant Citrobacter freundii Isolates in a Burn Hospital in Northeast of Iran: A Single-Center Cross-sectional Study. Res Mol Med.

[32] Praharaj AK, Khajuria A, Kumar M, Grover N (2016). Phenotypic detection and molecular characterization of beta-lactamase genes among Citrobacter species in a tertiary care hospital. Avicenna J Med.

[33] Arana DM, Ortega A, González-Barberá E, Lara N, Bautista V, Gómez-Ruíz D, Citrobacter C-R, spp. isolated in Spain from,  (2013). to 2015 produced a variety of carbapenemases including VIM-1, OXA-48, KPC-2, NDM-1 and VIM-2. J Antimicrob Chemother.

[34] Goossens H, Grabein B (2005). Prevalence and antimicrobial susceptibility data for extended-spectrum beta-lactamase- and AmpC-producing Enterobacteriaceae from the MYSTIC Program in Europe and the United States (1997–2004). Diagn Microbiol Infect Dis.

[35] Hawser SP, Bouchillon SK, Hoban DJ, Badal RE (2010). Epidemiologic trends, occurrence of extended-spectrum beta-lactamase production, and performance of ertapenem and comparators in patients with intra-abdominal infections: analysis of global trend data from 2002–2007 from the SMART study. Surg Infect.

[36] Ishii Y, Tateda K, Yamaguchi K (2008). Japan Antimicrobial Resistance Surveillance Participants Group (JARS). Evaluation of antimicrobial susceptibility for beta-lactams using the Etest method against clinical isolates from 100 medical centers in Japan (2006). Diagn Microbiol Infect Dis..

[37] Jones ME, Karlowsky JA, Draghi DC, Thornsberry C, Sahm DF, Nathwani D (2003). Epidemiology and antibiotic susceptibility of bacteria causing skin and soft tissue infections in the USA and Europe: a guide to appropriate antimicrobial therapy. Int J Antimicrob Agents.

[38] Logan LK, Renschler JP, Gandra S, Weinstein RA, Laxminarayan R (2015). Carbapenem-Resistant Enterobacteriaceae in Children, United States, 1999–2012. Emerg Infect Dis.

[39] Mylvaganam H, Kolstad H, Breistein RI, Lind G, Skutlaberg DH (2017). Extended spectrum cephalosporin resistance among clinical isolates of Enterobacteriaceae in West Norway during 2006–2013; a prospective surveillance study. APMIS Acta Pathol Microbiol Immunol Scand.

[40] Nishio H, Komatsu M, Shibata N, Shimakawa K, Sueyoshi N, Ura T (2004). Metallo-β-Lactamase-Producing Gram-Negative Bacilli: Laboratory-Based Surveillance in Cooperation with 13 Clinical Laboratories in the Kinki Region of Japan. J Clin Microbiol.

[41] Orrett FA, Shurland SM (2000). Prevalence of bacterial pathogens and susceptibility patterns from clinical sources in Trinidad. West Indian Med J.

[42] Räisänen K, Sarvikivi E, Arifulla D, Pietikäinen R, Forsblom-Helander B, Tarkka E (2021). Three clusters of carbapenemase-producing *Citrobacter freundii* in Finland, 2016–20. J Antimicrob Chemother.

[43] Shetty J, Kotigadde S (2007). Antibiotic sensitivity pattern of Citrobacter isolated from various clinical specimens in a tertiary care hospital. Indian J Pathol Microbiol.

[44] De Geyter D, Blommaert L, Verbraeken N, Sevenois M, Huyghens L, Martini H (2017). The sink as a potential source of transmission of carbapenemase-producing Enterobacteriaceae in the intensive care unit. Antimicrob Resist Infect Control.

[45] Entezari M, Karimi S, Ahmadieh H, Mahmoudi AH, Parhizgar H, Yaseri M (2016). A Large Outbreak of Fulminant Bacterial Endophthalmitis after Intravitreal Injection of Counterfeit Bevacizumab. Graefes Arch Clin Exp Ophthalmol.

[46] Gaibani P, Ambretti S, Farruggia P, Bua G, Berlingeri A, Tamburini MV (2013). Outbreak of Citrobacter freundii carrying VIM-1 in an Italian Hospital, identified during the carbapenemases screening actions, June 2012. Int J Infect Dis IJID Off Publ Int Soc Infect Dis.

[47] Gobeille Paré S, Mataseje LF, Ruest A, Boyd DA, Lefebvre B, Trépanier P (2020). Arrival of the rare carbapenemase OXA-204 in Canada causing a multispecies outbreak over 3 years. J Antimicrob Chemother.

[48] Jiménez A, Castro JG, Munoz-Price LS, de Pascale D, Shimose L, Mustapha MM (2017). Outbreak of *Klebsiella pneumoniae* Carbapenemase-Producing *Citrobacter freundii* at a Tertiary Acute Care Facility in Miami. Florida Infect Control Hosp Epidemiol.

[49] Jolivet S, Couturier J, Vuillemin X, Gouot C, Nesa D, Adam M (2021). Outbreak of OXA-48-producing Enterobacterales in a haematological ward associated with an uncommon environmental reservoir, France, 2016 to 2019. Eurosurveillance.

[50] Muta T, Tsuruta N, Seki Y, Ota R, Suzuki S, Shibata N, et al. A Nosocomial Outbreak Due to Novel CTX-M-2-Producing Strains of Citrobacter koseri in a Hematological Ward. 16495644

[51] Nada T, Baba H, Kawamura K, Ohkura T, Torii K, Ohta M (2004). A small outbreak of third generation cephem-resistant Citrobacter freundii infection on a surgical ward. Jpn J Infect Dis.

[52] Pletz MW, Wollny A, Dobermann U-H, Rödel J, Neubauer S, Stein C (2018). A Nosocomial Foodborne Outbreak of a VIM Carbapenemase-Expressing Citrobacter freundii. Clin Infect Dis.

[53] Rödel J, Mellmann A, Stein C, Alexi M, Kipp F, Edel B (2019). Use of MALDI-TOF mass spectrometry to detect nosocomial outbreaks of Serratia marcescens and Citrobacter freundii. Eur J Clin Microbiol Infect Dis Off Publ Eur Soc Clin Microbiol.

[54] Royer G, Fourreau F, Gomart C, Maurand A, Hacquin B, Ducellier D (2020). Outbreak of an Uncommon Rifampin-resistant blaNDM-1Citrobacter amalonaticus Strain in a Digestive Rehabilitation Center: The Putative Role of Rifaximin. Clin Infect Dis Off Publ Infect Dis Soc Am.

[55] Schweizer C, Bischoff P, Bender J, Kola A, Gastmeier P, Hummel M (2019). Plasmid-Mediated Transmission of KPC-2 Carbapenemase in Enterobacteriaceae in Critically Ill Patients. Front Microbiol.

[56] Segal E, Bar Yosef S, Axel A, Keller N, Shlaeffer F, Amir A, et al. Outbreak of sepsis following surgery: utilizing 16S RNA sequencing to detect the source of infection. Cureus. 14:e22487. 10.7759/cureus.22487 PMC8944214 35371778

[57] Jabeen I, Islam S, Hassan AKMI, Tasnim Z, Shuvo SR. A brief insight into Citrobacter species - a growing threat to public health. Front Antibiot. 2023;2. 10.3389/frabi.2023.1276982 PMC11731968 39816660

[58] Tacconelli E, Sifakis F, Harbarth S, Schrijver R, van Mourik M, Voss A (2018). Surveillance for control of antimicrobial resistance. Lancet Infect Dis.

[59] Global Antimicrobial Resistance and Use Surveillance System (GLASS) Report 2022-eng.pdf. https://iris.who.int/bitstream/handle/10665/364996/9789240062702-eng.pdf. Accessed 5 Feb 2024.

[60] Schwaber MJ, De-Medina T, Carmeli Y (2004). Epidemiological interpretation of antibiotic resistance studies - what are we missing?. Nat Rev Microbiol.

[61] Hamerlinck H, Aerssens A, Boelens J, Dehaene A, McMahon M, Messiaen A-S (2023). Sanitary installations and wastewater plumbing as reservoir for the long-term circulation and transmission of carbapenemase producing Citrobacter freundii clones in a hospital setting. Antimicrob Resist Infect Control.

[62] Kus JV, Burrows LL, Enna SJ, Bylund DB (2007). Infections due to Citrobacter and Enterobacter. xPharm: The Comprehensive Pharmacology Reference.

[63] Shih C-C, Chen Y-C, Chang S-C, Luh K-T, Hsieh W-C (1996). Bacteremia Due to Citrobacter Species: Significance of Primary Intraabdominal Infection. Clin Infect Dis.

