# Supplementary material for: Epidemiology of Citrobacter spp. infections among hospitalized patients: a systematic review and meta-analysis
Source: BMC Infect Dis. 2024 Jul 2;24:662. doi: 10.1186/s12879-024-09575-8 (PMC11221093; doi:10.1186/s12879-024-09575-8)
Supplement: Supplementary file 1 — Supplementary Material 1. [file 12879_2024_9575_MOESM1_ESM.docx]

**Additional file 1**

**Epidemiology of *Citrobacter* spp. infections among hospitalized patients: A systematic review and meta-analysis**

**Table of contents**

[**Review definitions** 2](#_Toc170302997)

[**Search strategy** 3](#_Toc170302998)

[Figure S1: Number of patients with *Citrobacter* infection/colonization reported in observational studies over time (n=15) 4](#_Toc170302999)

[Figure S2: Included *Citrobacter* isolates by specimen type and study category 5](#_Toc170303000)

[Table S1 : Countermeasures implemented to control the spread of *Citrobacter* spp. during hospital outbreaks (n=13 outbreaks) 6](#_Toc170303001)

[Figure S3: Summary plot for quality of reporting in the included outbreak studies based on ORION statement 7](#_Toc170303002)

[Figure S4 : Traffic light plot of risk of bias in Cohort studies (n=10) 8](#_Toc170303003)

[Figure S5 : Summary plot for risk of bias in Cohort studies (n=10) 9](#_Toc170303004)

[Figure S6 : Traffic light plot of risk of bias in cross-sectional studies (n=4) 10](#_Toc170303005)

[Figure S7 : Summary plot of risk of bias in cross-sectional studies (n=4) 10](#_Toc170303006)

## **Review definitions**

- *Citrobacter* outbreak report was defined as a published outbreak study in peer-reviewed literature reporting a hospital outbreak in which *Citrobacter* spp. infection and/or colonization was responsible for at least 1/3 of the cases included in the outbreak. In addition, the epidemiological and clinical characteristics of patients involved in the outbreak had to be described. Experimental studies relying only on microbiological analysis with no epidemiological evaluation were excluded.
- Surveillance studies were defined as studies using laboratory data collected as part of routine work, including minimal data collection on each case, and lacking a clear study hypothesis.
- Antibiotic resistance percentages were defined as the number of *Citrobacter* isolates resistant and/or intermediate to the antibiotic class of interest divided by the total number of *Citrobacter* isolates tested.

## **Search strategy**

**PubMed**

("citrobacter"[MeSH Terms] OR "citrobacter" OR "C? freundii" OR "C?koseri" OR "C?braakii") **AND** ("cross infection"[MeSH Terms] OR "nosocomial infection" OR "hospital?acquired" OR "healthcare?acquired" OR "disease outbreaks"[MeSH Terms] OR "outbreak" OR "surveillance" OR "Hospitalised" OR "Hospitalized" OR "inpatients"[MeSH Terms] OR "inpatient") AND (("2000/01/01"[Date - Publication] : "3000"[Date - Publication]))

**EMBASE**

('citrobacter'/exp OR 'citrobacter' OR 'citrobacter freundii'/exp OR 'citrobacter freundii' OR 'citrobacter koseri'/exp OR 'citrobacter koseri' OR 'citrobacter braakii'/exp OR 'citrobacter braakii' OR 'c#freundii' OR 'c#koseri' OR 'c#braakii') AND ('hospital infection'/exp OR 'hospital infection' OR 'outbreak'/exp OR 'outbreak' OR 'surveillance'/exp OR surveillance OR 'nosocomial transmission'/exp OR 'nosocomial transmission' OR 'inpatient'/exp OR 'inpatient' OR hospitalized OR 'healthcare associated infection'/exp OR 'healthcare associated infection') AND [humans]/lim AND [2000-2023]/py

## Figure S1: Number of patients with *Citrobacter* infection/colonization reported in observational studies over time (n=15)

Prior to 2010, 7 studies were included, from 2010 to 2019, we included 5 studies, and 3 studies were included from 2020 to 2023.

## Figure S2: Included *Citrobacter* isolates by specimen type and study category

Other specimens: pus, skin and soft tissue, abscess, drain hole, peritoneal-fluid, intra-abdominal fluid, ear secretion, ophthalmic secretion.

## Table S1 : Countermeasures implemented to control the spread of *Citrobacter* spp. during hospital outbreaks (n=13 outbreaks)

|  | Royer et al. (2020) - France | Nada et al. (2004) - Japan | Entezari et al. (2016) - Iran | Muta et al. (2006) - Japan | Segal et al. (2022) - Israel | Gobeille Pare et al. (2020) - Canada | De Geyter et al. (2017) - Belgium | Schweizer et al (2019) - Gemany | Gaibani et al. (2013) - Italy | Jolivet et al. (2021) - France | Jimenez et al. (2017) - USA | Pletz et al. (2018)- Germany | Rodel et al. (2019) -Germany |
| --- | --- | --- | --- | --- | --- | --- | --- | --- | --- | --- | --- | --- | --- |
| Patient-targeted interventions | | | | | | | | | | | | | |
| Isolation or cohorting of colonized/infected patients | **x** |  |  |  |  | **x** | **x** |  | **x** | **x** | **x** | **x** |  |
| Strict contact precautions (PPE) | **x** | **x** |  |  |  | **x** |  |  |  | **x** | **x** |  |  |
| Active patient surveillance | **x** |  |  |  |  | **x** |  |  | **x** | **x** | **x** | **x** | **x** |
| Dedicated staff | **x** |  |  |  |  | **x** |  |  |  | **x** |  |  |  |
| Contact tracing |  |  |  |  |  |  |  |  |  | **x** |  |  |  |
| Daily bathing with 2% chlorhexidine-impregnated wipes of all inpatients |  |  |  |  |  |  |  |  |  |  | **x** |  |  |
| Staff targeted interventions | | | | | | | | | | | | | |
| Hand hygiene reinforcement |  |  |  | **x** |  |  | **x** |  | **x** | **x** | **x** |  |  |
| Education and training of staff |  |  |  |  |  |  | **x** |  | **x** | **x** | **x** | **x** | **x** |
| Reinforcement of infection control practices | **x** |  |  |  |  | **x** |  |  |  | **x** |  |  |  |
| Antimicrobial stewardship program |  |  |  | **x** |  | **x** | **x** |  |  |  |  |  |  |
| Reporting to hospital management or health authorities |  |  |  |  |  |  |  |  |  |  | **x** | **x** |  |
| Screening of staff |  |  |  |  |  |  |  |  |  |  |  | **x** |  |
| Environmental interventions |  |  |  |  |  |  |  |  |  |  |  |  |  |
| Environmental cleaning/disinfection (ex : sinks, toilets, patient’s room) |  |  |  | **x** |  | **x** | **x** | **x** | **x** | **x** | **x** | **x** |  |
| Replacement of toilets bowls /siphons or others environmentals materials |  |  |  |  |  |  | **x** | **x** |  | **x** | **x** |  | **x** |

**PPE : Personal Protective Equipment**

Figure S3: Summary plot for quality of reporting in the included outbreak studies based on ORION statement

## Figure S4 : Traffic light plot of risk of bias in Cohort studies (n=10)


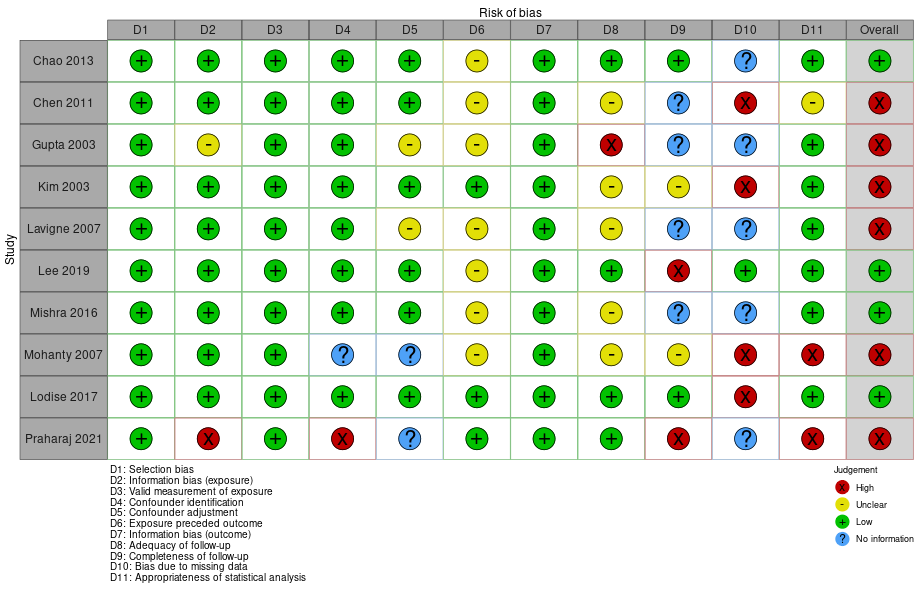


## Figure S5 : Summary plot for risk of bias in Cohort studies (n=10)

## Figure S6 : Traffic light plot of risk of bias in cross-sectional studies (n=4)


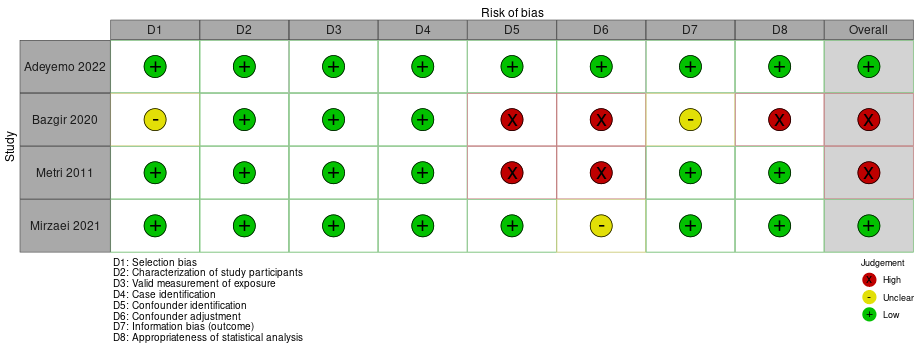


## Figure S7 : Summary plot of risk of bias in cross-sectional studies (n=4)
