# Supplementary material for: Epidemiology of Citrobacter spp. infections among hospitalized patients: a systematic review and meta-analysis
Source: BMC Infect Dis. 2024 Jul 2;24:662. doi: 10.1186/s12879-024-09575-8 (PMC11221093; doi:10.1186/s12879-024-09575-8)
Supplement: Supplementary file 2 — Supplementary Material 2. [file 12879_2024_9575_MOESM2_ESM.docx]

**Additional file 2**

**Epidemiology of *Citrobacter* spp. infections among hospitalized patients: A systematic review and meta-analysis**

**Table of contents**

[Figure S1: Pooled resistance-percentage for ESBL-producing *Citrobacter* isolates in all *Citrobacter* isolates 2](#_Toc158107639)

[Figure S2: Pooled resistance-percentage for AmpC-producing *Citrobacter* isolates in all *Citrobacter* isolates 3](#_Toc158107640)

[Figure S3: Pooled resistant percentages to imipenem in *Citrobacter* isolates 4](#_Toc158107641)

[Figure S4: Pooled resistant percentages to meropenem in *Citrobacter* isolates 5](#_Toc158107642)

[Figure S5: Pooled resistant percentages to ceftazidime in *Citrobacter* isolates 6](#_Toc158107643)

[Figure S6: Pooled resistant percentages to cefotaxime in *Citrobacter* isolates 7](#_Toc158107644)

[Figure S7: Pooled resistant percentages to piperacillin-tazobactam in *Citrobacter* isolates 8](#_Toc158107645)

[Figure S8: Pooled resistant percentages to amikacin in *Citrobacter* isolates 9](#_Toc158107646)

[Figure S9 : Pooled resistant percentages to gentamicin in *Citrobacter* isolates 10](#_Toc158107647)

[Figure S10: Pooled resistant percentages to ciprofloxacin in *Citrobacter* isolates 11](#_Toc158107648)

[Figure S11: Pooled resistant percentages to co-trimoxazole in *Citrobacter* isolates 12](#_Toc158107649)

# Figure S1: Pooled resistance-percentage for ESBL-producing *Citrobacter* isolates in all *Citrobacter* isolates


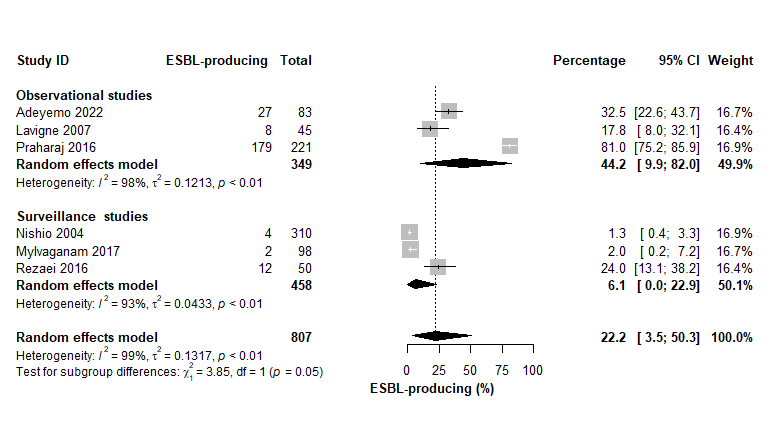


# Figure S2: Pooled resistance-percentage for AmpC-producing *Citrobacter* isolates in all *Citrobacter* isolates


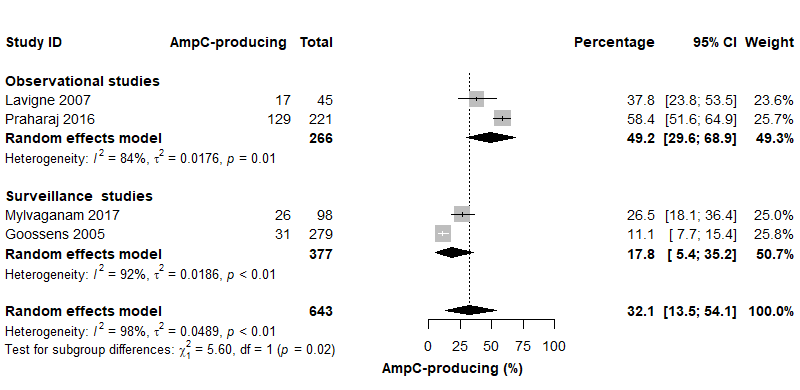


# Figure S3: Pooled resistance percentages to imipenem in *Citrobacter* isolates


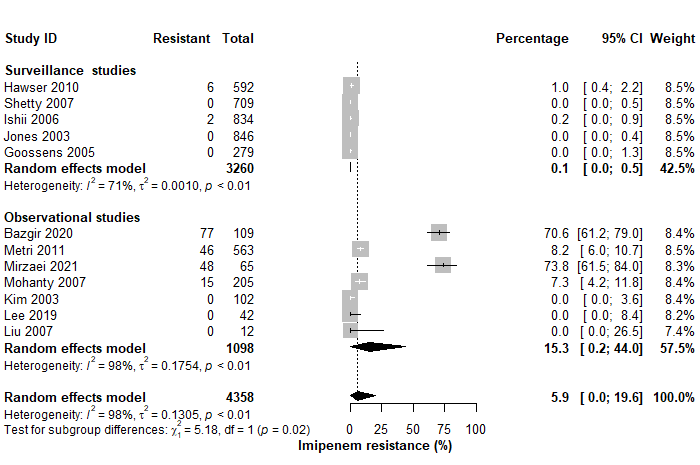


# Figure S4: Pooled resistance percentages to meropenem in *Citrobacter* isolates


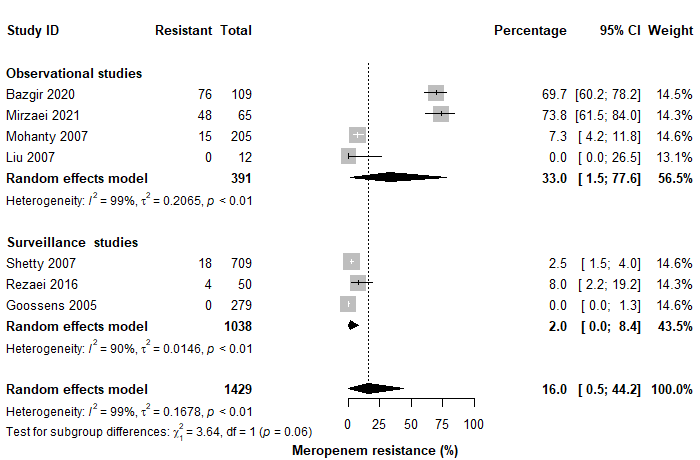


Figure S5: Pooled resistance percentages to ceftazidime in *Citrobacter* isolates


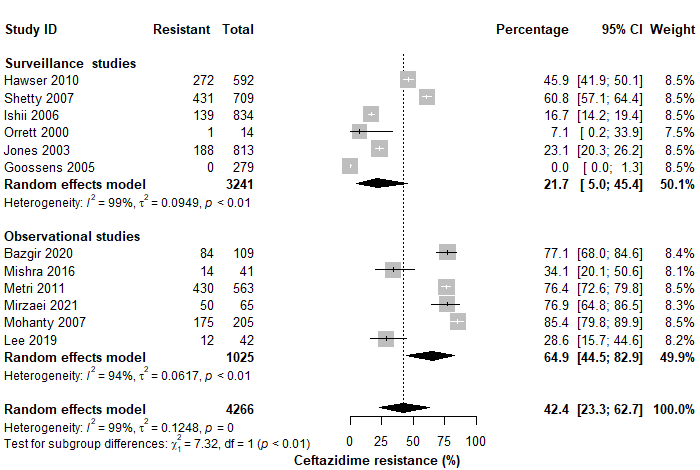


# Figure S6: Pooled resistance percentages to cefotaxime in *Citrobacter* isolates

# Figure S7: Pooled resistance percentages to piperacillin-tazobactam in *Citrobacter* isolates

Figure S8: Pooled resistance percentages to amikacin in *Citrobacter* isolates


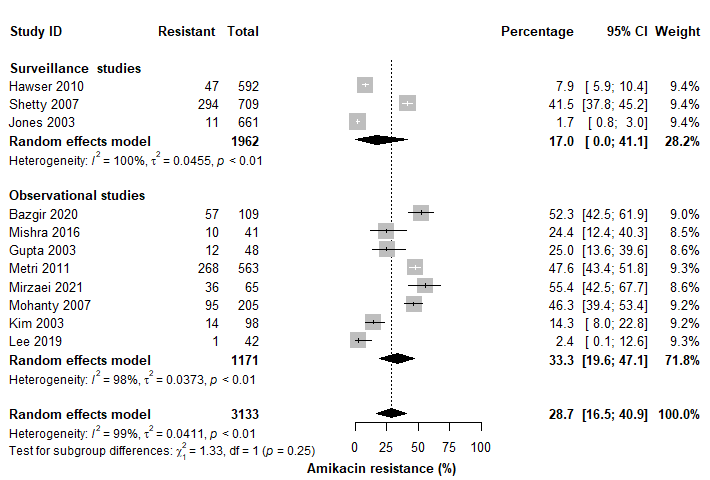


Figure S9 : Pooled resistance percentages to gentamicin in *Citrobacter* isolates

# Figure S10: Pooled resistance percentages to ciprofloxacin in *Citrobacter* isolates

# Figure S11: Pooled resistance percentages to co-trimoxazole in *Citrobacter* isolates
